# Laboratory method of microbial induced solidification/stabilization for municipal solid waste incineration fly ash

**DOI:** 10.1016/j.mex.2019.05.006

**Published:** 2019-05-07

**Authors:** Hui Xu, Hao Zheng, Jin-nan Wang, Xiao-qing Ding, Ping Chen

**Affiliations:** School of Civil Engineering and Architecture, Zhejiang Sci-Tech University, Hangzhou, 310018, China

**Keywords:** Microbial induced solidification/stabilization method, Microbial induced carbonate precipitation (MICP), Solidification/stabilization method, Municipal solid waste incineration (MSWI) fly ash, Endogenous calcium, Heavy metal leachability, Compressive strength

## Abstract

This paper presents a method to solidify/stabilize the municipal solid waste incineration (MSWI) fly ash by originally employing the microbial induced carbonate precipitation (MICP) technique. In this method, the rich endogenous calcium in the MSWI fly ash was utilized to induce calcite precipitation, which is different from the operation of adding extra calcium source in previous researches. The fly ash sample had a CaO content of 44.5%, and its leaching concentrations of Zn, Cr and Pb exceed the limits of the identification standard for hazardous wastes in China. The optical density at 600 nm (OD600) of the bacterial solution was about 1.0 after the processes of bacterial activation and reproduction. The prepared fly ash sample was well mixed with bacterial solution at an ash-liquid ratio of 1 kg: 0.3 L and cured at a temperature of 20 °C and a humidity of ≥95% for 7 days. After treatment, the heavy metal leachability significantly reduced to meet the standard for pollution control of landfill site, and the unconfined compressive strength increased approximately 40%. The precipitated carbonates were verified by SEM-EDS analysis and quantified by measurement of carbonate content via acid-dissolving method. The results shone a light on the possibility of using MICP technique as a useful and efficient tool to stabilize the MSWI fly ash before being reused or properly stored in landfills.

• The MICP method is efficient to reduce the heavy metal leachability and increase the compressive strength of MSWI fly ash.
• The endogenous calcium in MSWI fly ash was utilized to induce calcite precipitation.
• The heavy metals in MSWI fly ash were well immobilized by the formation of carbonates.

## Graphical abstract

**Figure:**
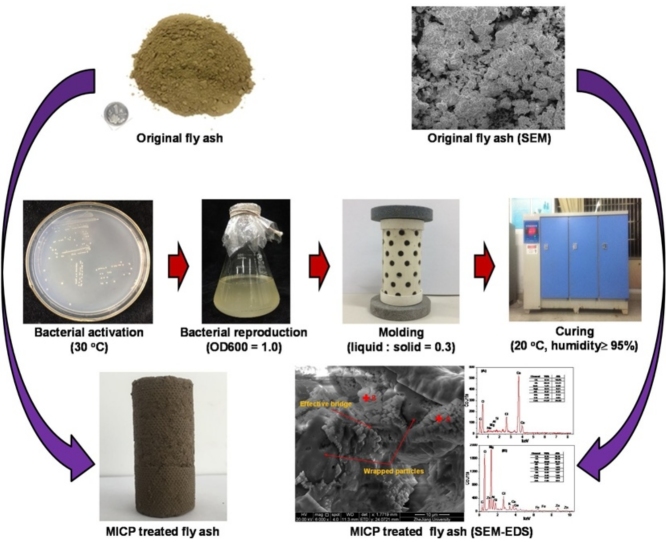

**Specifications Table**

**Table:** 

Subject area:	Environmental Science
More specific subject area:	Management of solid waste
Method name:	Microbial induced solidification/stabilization method
Name and reference of original method:	Derived from: J. K. Mitchell, J. C. Santamarina, Biological considerations in geotechnical engineering, J. Geotech. Geoenviron. Eng. 131(2005)1222–1233.

## Methodology background

The municipal solid waste incineration (MSWI) fly ash is a kind of porous material and rich in inorganic substances such as heavy metals, calcium, etc. MSWI fly ash is classified as hazardous waste due to its high content of heavy metals. According to the existing legislation of most countries, proper treatment must be taken for the MSWI fly ash before its disposal in a sanitary landfill [1].

Microbial induced carbonate precipitation (MICP) is a recently developed new technique and widely used in geotechnical engineering [2,3]. The MICP process involves two main stages: urea hydrolysis and carbonate precipitation [[4], [5], [6]], as shown in Eqs. (1) and (2). In most researches, the solution contained urease-producing bacteria, calcium chloride, urea, nutrient, etc., was added into the soil column to form calcium carbonate that binds the soil particles together, improving the engineering properties of soils [[7], [8], [9], [10]].

(1) $$CO{\left(NH_{2}\right)}_{2}+2H_{2}O\overset{Urease}{⟶}CO_{3}^{2-}+2NH_{4}^{+}$$

(2) $$M^{a+}+CO_{3}^{2-}\to M_{2}{\left(CO^{3}\right)}_{a}$$

This method originally employs the MICP technique to stabilize the MSWI fly ash, i.e., reduce the heavy metal leachability and enhance the compression strength. This method utilizes the rich calcium embodied in the MSWI fly ash, which is different from the operations adopted by the previous researches, i.e., adding extra calcium source.

## Method details

### Preparation of MSWI fly ash

**Fig. 1:**
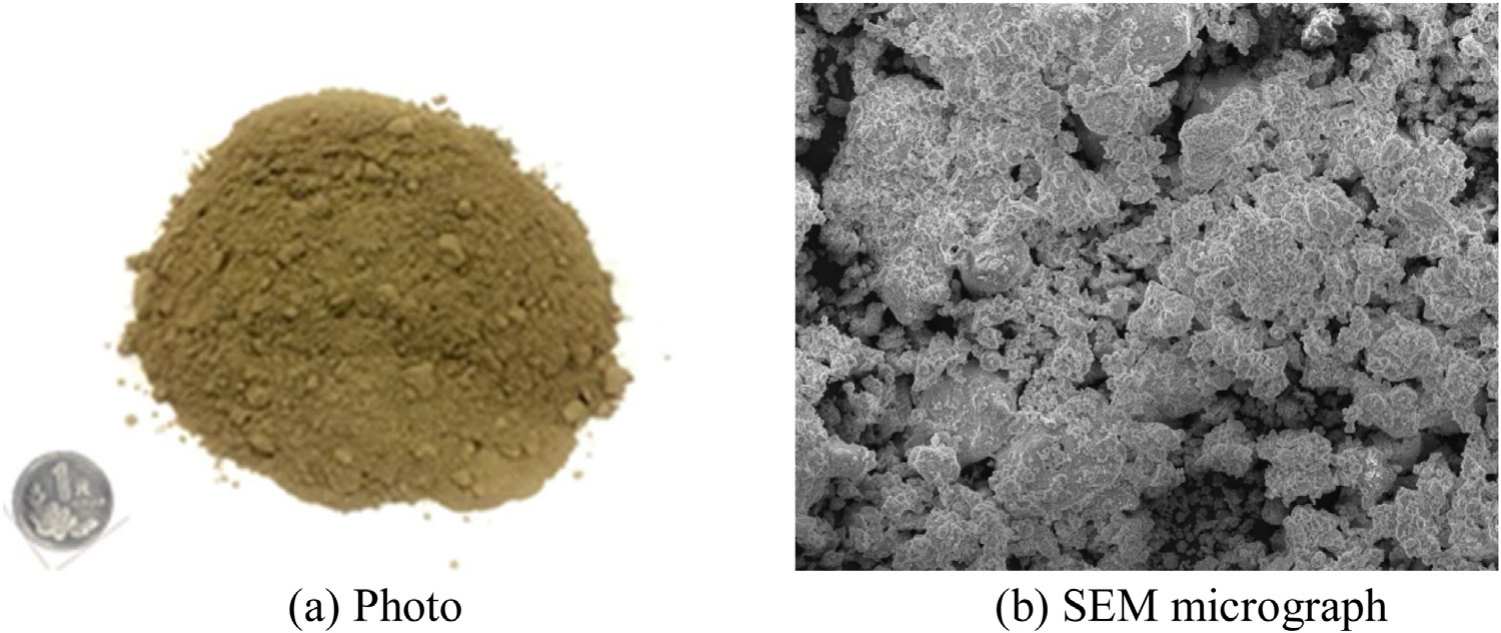
MSWI fly ash.

The MSWI fly ash was sampled from a MSWI plant in Hangzhou, China. The morphological structure was analyzed by an SEM-EDS equipment (FEI Quanta 650 FEG ESEM, U.S). As shown in Fig. 1, the original fly ash was a kind of porous structure material with particle sizes varied in a wide range, from smaller than 1 μm to larger than 100 μm. The morphology of original fly ash was irregular in shape and contained many pores. To obtain the physicochemical properties of the MSWI fly ash, the following tests were carried out. The particle size distribution was analyzed by the combination of mechanical sieve method (diameter≥0.075 mm) and hydrometer method (diameter<0.075 mm). The chemical compositions were determined by an energy dispersive X-ray spectrometer (EDXS, JSM-5610LV, Japan). The toxicity characteristic leaching procedure (TCLP) test was conducted following China EPA method HJ557-2009 to obtain the leaching concentration of trace metals. In the leaching procedure, 100 g dry fly ash sample was well mixed with 1 L deionized water in a polyethylene bottle. After that, the mixture was vibrated at a frequency of 110 ± 10 times/min and an amplitude of 40 mm and temperature of 23 ± 2 °C for 8 h. The resulting solution filtered through a membrane disk was analyzed for metal content by using an inductively coupled plasma-mass spectrometer (ICP-MS, Agilent 7700X, China).

The test results are presented in Table 1. The sand, silt and clay contents were 14.5%, 69.0% and 15.8%, respectively, indicating that the particles were more like silt. The particles were poorly graded with nonuniform coefficient *C_c_* = 28.5 and curvature coefficient *C_u_* = 14.6. The content of CaO was as high as 44.5%, hence, there was a sufficient supply of endogenous Ca for MICP treatment. The high content of alkaline oxides resulted in a strong alkalinity of the fly ash, and the pH value was 10.8. The leaching concentrations of Zn, Cr and Pb exceed the limits of the identification standard for hazardous wastes in China, as shown in Fig. 6 in the following section. Therefore, stabilization of heavy metals in the MSWI fly ash is necessary whether they are going to be reused or properly stored in landfills.

**Table 1:** Physicochemical properties of MSWI fly ash.

Particle distribution	Sand content		Silt content	Clay content	*C_c_*	*C_u_*
	14.5±2.5%		69.0±2.3%	15.8±1.3%	28.5	14.6
Chemical composition (%)	CaO		SiO_2_	Al_2_O_3_	MgO	Na_2_O
	44.5±6.6		12.2±0.9	4.8±0.5	1.9±0.4	4.7±0.5
Heavy metal leachability (mg/L)	Ca	Zn	Cu	Pb	Cr	Cd
	1852.4±43.4	108.3±9.l	9.3±1.3	7.4±0.9	10.8±0.6	0.46±0.07

### Preparation of bacterial solution

**Fig. 2:**
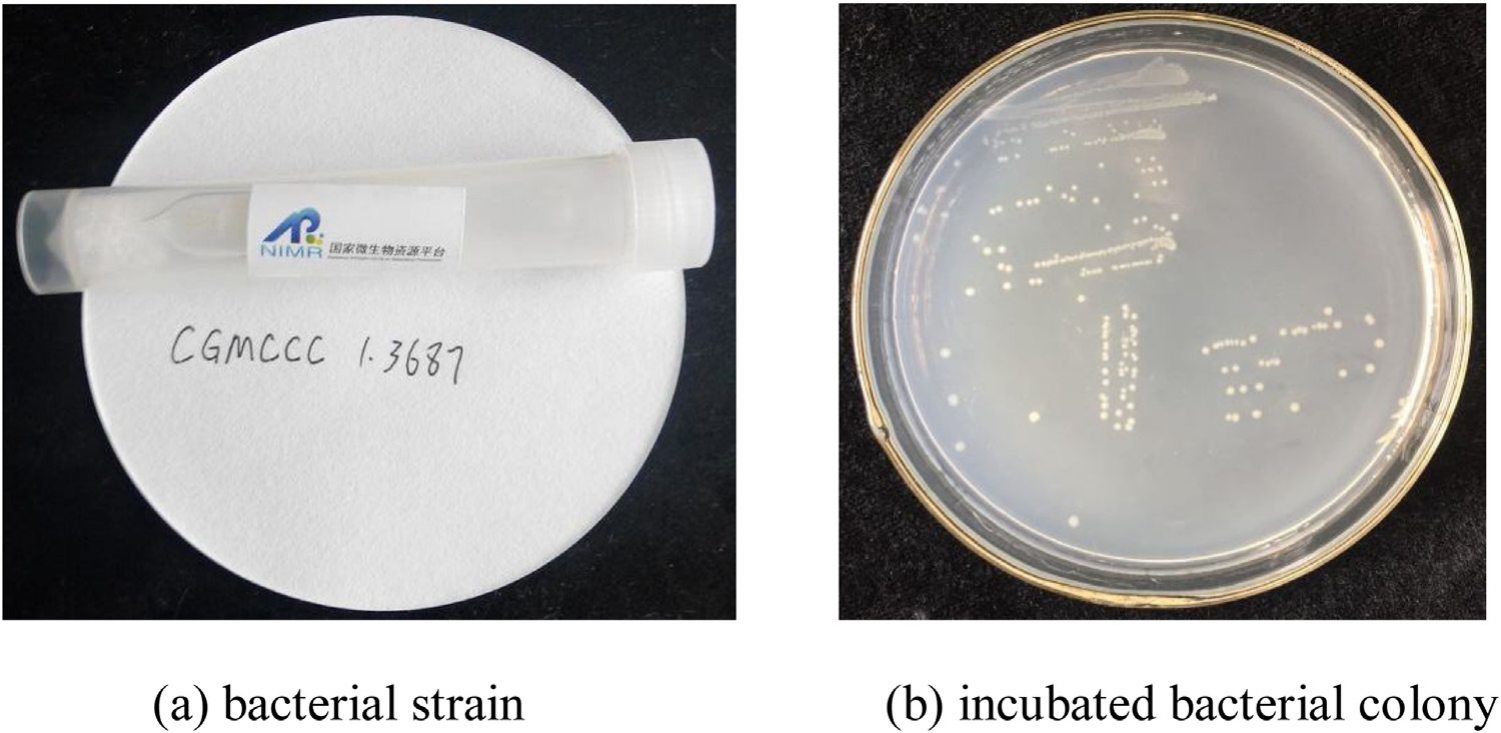
Bacterial strain and incubated bacterial colony.

*S. pasteurii* (CGMCC1.3687), a high-performance urease-producing strain, was adopted in this study, as shown in Fig. 2(a). This bacterial strain was rehydrated under a sterile condition. Then, the rehydrated strain was removed to be grown in the solid medium of a Petri dish by streaking in laminar flow hood to avoid contamination. The solid medium was composed of 5 g/L peptone, 3 g/L beef extract, 20 g/L urea and 15 g/L agar, at a pH value of about 6.8, which supplied the necessary carbon, nitrogen, and other ingredients for the bacteria to survive. After incubation at temperature of 30 °C for 24 h, several bacterial colonies formed in the solid medium and can be visually identified, as shown in Fig. 2(b), indicating that the bacterial activation was succeeded.

**Fig. 3:**
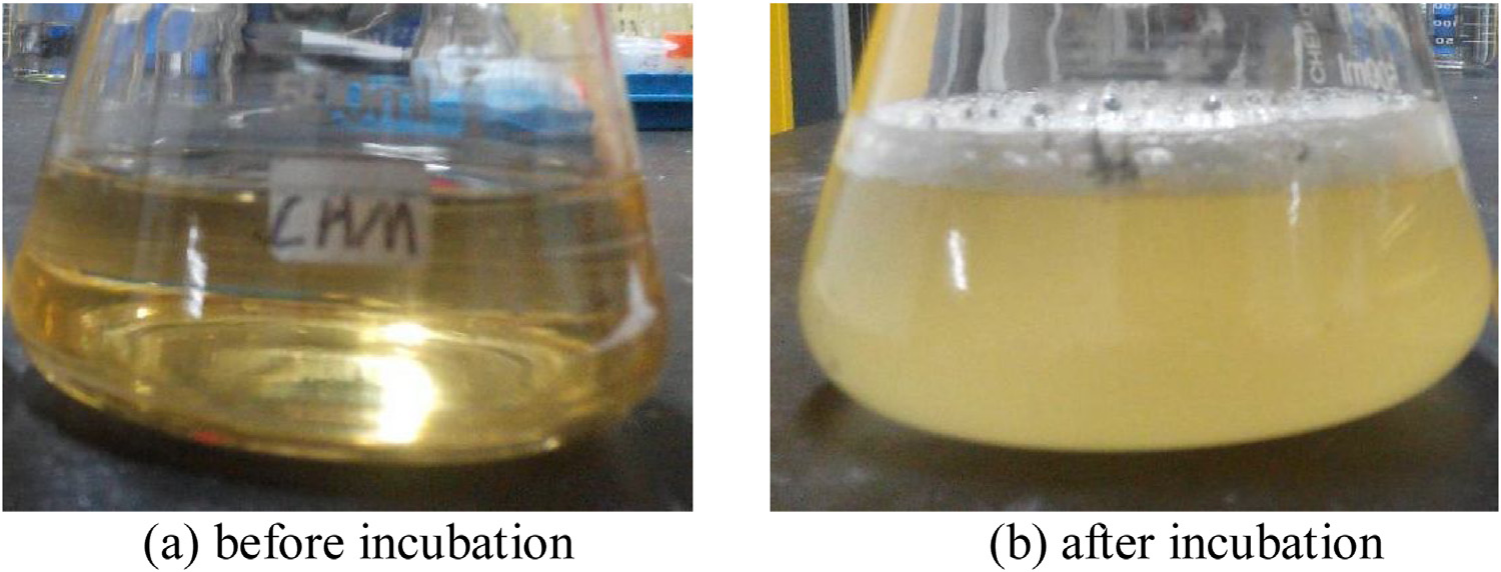
Bacterial solution.

The activated bacterial colony was selected and removed to an Erlenmeyer flask which contained 50 ml solution medium under a sterile condition, as shown in Fig. 3(a). The solution medium was made of 5 g peptone, 3 g beef extract and 20 g urea per 1 L distilled water, and the pH value was about 7.0. After that, the bacterial solution was placed in an incubator at shaking rate of 220 rpm and temperature of 30 °C for bacterial reproduction. After 24 h of incubation, the solution medium became muddy, as shown in Fig. 3(b), due to a mass propagation of the bacteria in the flask. Subsequently, the bacterial solution was placed for one hour and sampled the supernatant liquid. The sample was analyzed for the OD600 value (the optical density at 600 nm) by using an ultraviolet-visible spectrophotometer. OD600 value is an indicator of bacterial concentration in solution, which could be used to describe the activity of *S.pasteurii*. If the tested OD600 value is higher than 0.15 [11], the bacterial solution is suitable and ready for the following operation.

### MICP treatment of MSWI fly ash

**Fig. 4:**
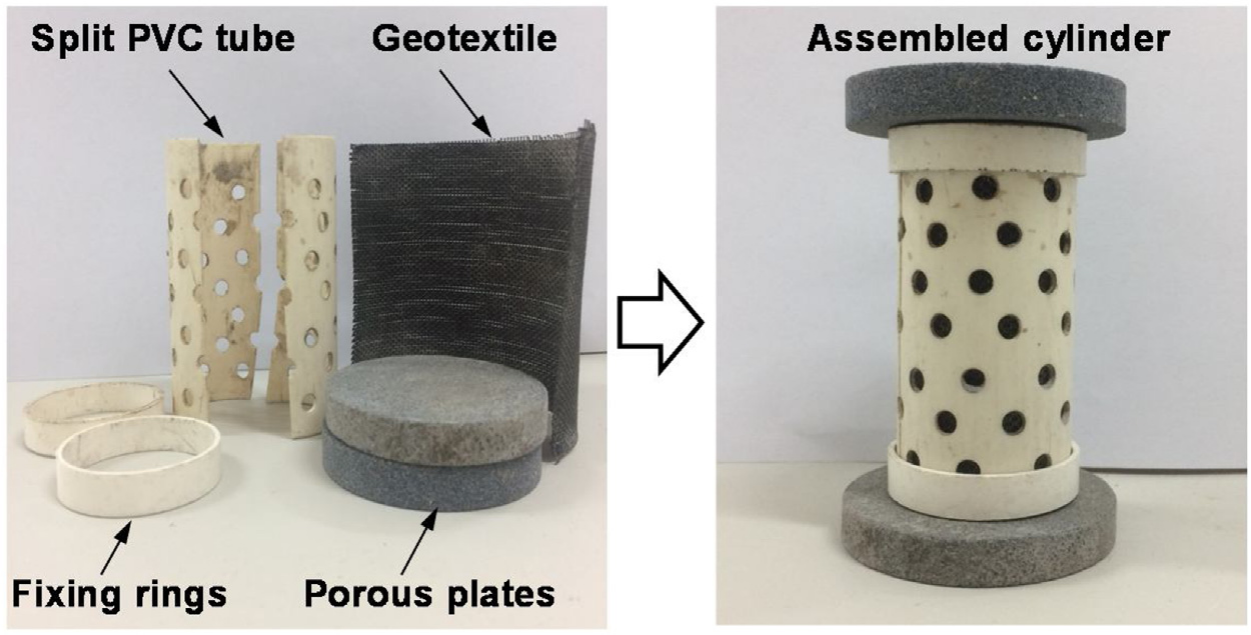
Moulding cylinder for MICP treatment.

The MSWI fly ash sample was dried at the temperature of 70 °C, and then mixed with the above bacterial solution at solid-liquid ratio of 1:0.3. After that, 120 g of the mixture was filled into a self-designed moulding cylinder. As shown in Fig. 4, the moulding cylinder consists of a split PVC tube with inner diameter of 36 mm and height of 80 mm, a layer of geotextile lined between the mixture and the PVC tube, and two porous plates placed on the bottom and top of the PVC tube, respectively. The geotextile and perforated PVC tube were installed to inlet adequate oxygen for microbial activity. After filling and compacting of the mixture, the surface was carefully levelled and the top fixing ring and porous plate were then assembled. Finally, the moulding cylinder was placed in an incubator with a temperature of 20 ± 2 °C and a humidity of ≥95% for curing. After curing for 24 h, the moulding cylinder was removed, and then the moulded sample column continued curing for 6 days.

### Investigation of solidification/stabilization effect

**Fig. 5:**
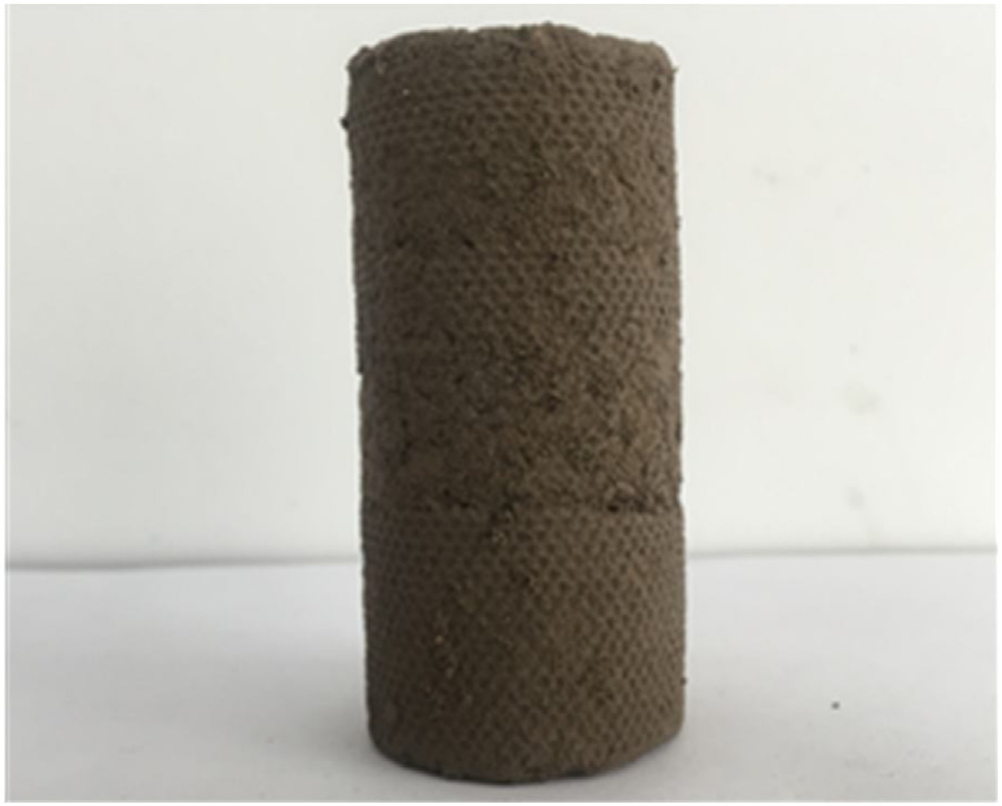
MSWI fly ash column after MICP treatment.

After curing, the solidified/stabilized sample was taken out, as shown in Fig. 5. The following tests were carried out to investigate the microbial induced solidification/ stabilization effect on MSWI fly ash. The unconfined compression test was conducted on the column sample by using a servo mechanical press. After that, the sample was collected and oven dried at temperature of 105±5°C for 24 h, and subsequently pulverized by a rubber mallet. Subsequently, the TCLP test was conducted to evaluate the heavy metal leachability of the solidified/stabilized sample by using the same method adopted for original fly ash sample. The SEM-EDS analysis was conducted to characterize the crystal shape and elemental composition of precipitated carbonate, and the bonding behavior between the grain host and biocemented agent. The precipitated carbonate content of the solidified/stabilized sample was determined by using an acid-dissolving method. 5–10 g dry crushed sample was added into a container filled with 2 mol/L hydrochloric acid solution, and the volume of CO_2_ gas was then measured using a U-tube manometer under standard conditions of 25 °C at 101.325 kPa [12]. The carbonate content was calculated based on the volume of CO_2_ gas. It should be noted that the carbonate content of the original MSWI fly ash was also measured, the difference between the amounts before and after MICP treatment is considered to be the precipitated carbonate. In addition, for comparison, the unconfined compression test was also conducted on the MSWI fly ash sample that was solely mixed with equal amount of deionized water, which was regarded as the control group.

**Fig. 6:**
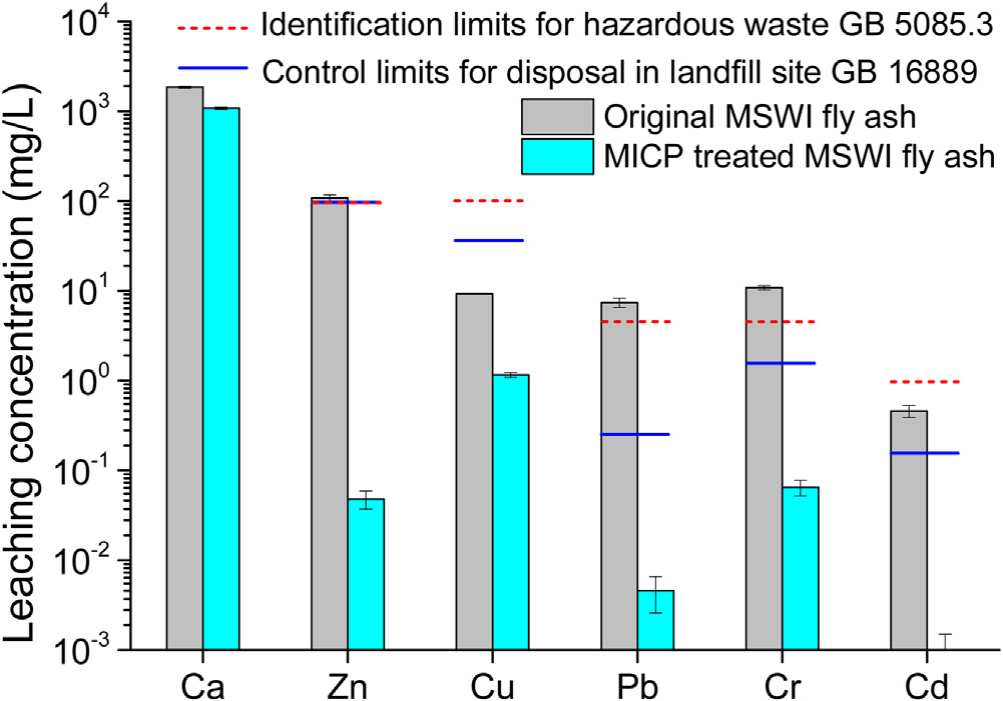
Leaching concentration of heavy metals of MICP treated sample.

### Method validation

The unconfined compressive strength of MICP treated sample reached as 0.156 ± 0.011 MPa, which was nearly 40% higher than the control group (0.112 ± 0.009 MPa). It indicated that the MSWI fly ash after MICP treatment has a considerable improvement in compressive strength.

The average leaching concentrations of Ca, Zn, Cu, Pb, Cr and Cd in the MICP treated sample were 1080.5, 0.048, 1.16, 0.005, 0.065 and 0.001 mg/L, respectively, being much lower than those of the original fly ash (Fig. 6). It is observed that the leaching concentrations of heavy metals were all constrained within the identification standard limits for hazardous wastes (GB5085.3) and in line with the standard for pollution control on the landfill site of municipal solid waste (GB16889) in China. This result indicated that the MICP treated MSWI fly ash was no longer identified as hazardous waste and can be properly disposed at MSW landfills.

When assuming that the reduction amount of leachable heavy metals in the MICP treated sample were totally transformed into carbonates, the content of the produced carbonates can be calculated via the following equation:

(3) $$n=\underset{i=1}{\Sigma }\frac{C_{bi}-C_{ai}}{M_{i}}$$

where *n* is the content of the produced carbonates, mol/g; *C_bi_* and *C_ai_* refer to the leaching concentrations of heavy metal *i* before and after MICP treatment respectively, g/g; *M_i_* is the molar mass of heavy metal *i*, g/mol. Based on the above method, the amount of the produced carbonates were calculated as 2.13 × 10^−4^ mol/g, which was much lower than that obtained from the acid-dissolving method, i.e., 5.29 × 10^−4^ mol/g (the carbonate content in original sample was deducted). The possible reason was that the available heavy metals might be much higher than those obtained from the TCLP test on original sample, for some kinds of heavy metals may continually dissolve out during the MICP process. Anyway, the conclusion is clear, which can be drawn that a mass of carbonates were newly produced in the MICP treated MSWI fly ash.

**Fig. 7:**
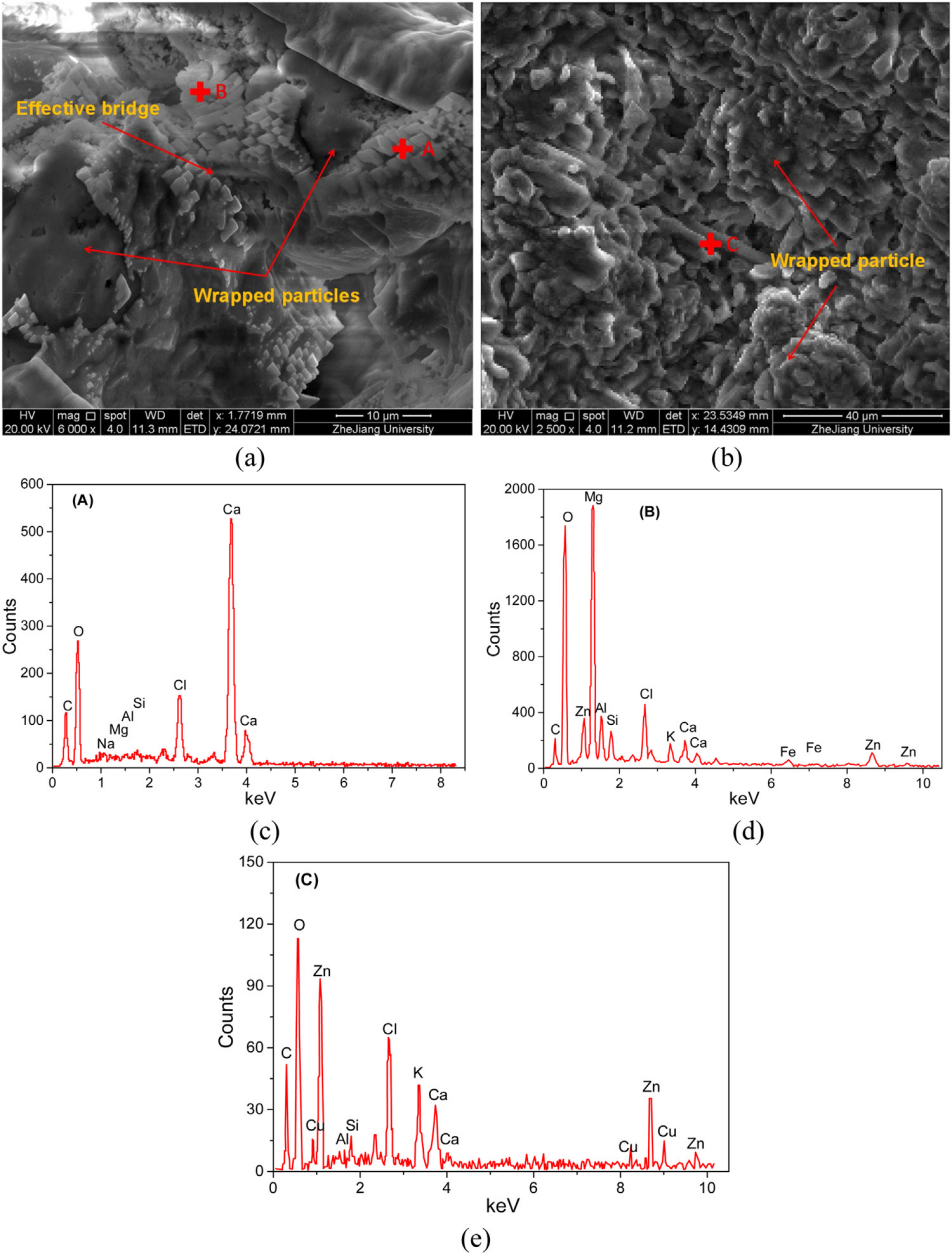
SEM and EDS images of MICP treated sample.

The SEM and EDS images of MICP treated sample is shown in Fig. 7. It is seen from the SEM micrographs that a significant amount of crystals was formed and wrapped at the surface of fly ash particles. The crystals formed effective bridges to link the neighboring particles together. As a result, the compressive strength enhanced. A mass of hexahedral crystals was observed to form in the particle voids (Fig. 7a, marked “A”). The EDS analysis illustrated that the major constituents for this crystal were Ca, O and C, and the atomic percent were 24.82%, 16.05% and 51.45%, respectively (Fig. 7c). Therefore, this crystal was probably the calcium carbonate. The cylindrical crystals observed in Fig. 7(a) (marked “B”) were mainly composed of Mg, O and C. From the perspective of atomic percent, Mg: C: O = 19.31:16.34:49.38 (Fig. 7d), which was close to the chemical formula of magnesium carbonate, hence there was a certain possibility for the presence of magnesium carbonate. Similarly, the needle-like crystals observed in Fig. 7(b) (marked “C”) were possibly zinc carbonate. These results indicated that there is a high possibility that the heavy metals contained in fly ashes transformed into carbonates after MICP treatment. The carbonates have stronger chemical stability, and therefore, lower leachability, in comparison to heavy metal ions. As a result, the leachable heavy metals can be strongly immobilized, and therefore the leaching toxicity significantly reduced.
